# The *RSPH4A* Gene in Primary Ciliary Dyskinesia

**DOI:** 10.3390/ijms24031936

**Published:** 2023-01-18

**Authors:** Wilfredo De Jesús-Rojas, Jesús Meléndez-Montañez, José Muñiz-Hernández, André Marra-Nazario, Francisco Alvarado-Huerta, Arnaldo Santos-López, Marcos J. Ramos-Benitez, Ricardo A. Mosquera

**Affiliations:** 1 Department of Pediatrics and Basic Science, Ponce Health Sciences University, Ponce, PR 00716, USA; 2 School of Medicine, University of Puerto Rico, Medical Sciences Campus, San Juan, PR 00921, USA; 3 Department of Pediatrics, McGovern Medical School, University of Texas Health Science Center at Houston, Houston, TX 77030, USA

**Keywords:** *RSPH4A*, primary ciliary dyskinesia, cilia, genotype-phenotype relationships, transmission electron microscopy, nasal nitric oxide, immunofluorescence, high-speed video microscopy analysis

## Abstract

The radial spoke head protein 4 homolog A (*RSPH4A*) gene is one of more than 50 genes that cause Primary ciliary dyskinesia (PCD), a rare genetic ciliopathy. Genetic mutations in the *RSPH4A* gene alter an important protein structure involved in ciliary pathogenesis. Radial spoke proteins, such as RSPH4A, have been conserved across multiple species. In humans, ciliary function deficiency caused by *RSPH4A* pathogenic variants results in a clinical phenotype characterized by recurrent oto-sino-pulmonary infections. More than 30 pathogenic *RSPH4A* genetic variants have been associated with PCD. In Puerto Rican Hispanics, a founder mutation (*RSPH4A* (c.921+3_921+6delAAGT (intronic)) has been described. The spectrum of the *RSPH4A* PCD phenotype does not include laterality defects, which results in a challenging diagnosis. PCD diagnostic tools can combine transmission electron microscopy (TEM), nasal nitric oxide (nNO), High-Speed Video microscopy Analysis (HSVA), and immunofluorescence. The purpose of this review article is to provide a comprehensive overview of current knowledge about the *RSPH4A* gene in PCD, ranging from basic science to human clinical phenotype.

## 1. Introduction

Primary ciliary dyskinesia (PCD) is a rare autosomal recessive ciliopathy characterized by abnormal functioning of motile cilia in the upper and lower respiratory epithelia [1]. Four key clinical features describe the PCD clinical phenotype: unexplained neonatal respiratory distress in a term infant, chronic wet cough, nasal congestion since six months of age, and abnormalities of organ left-right asymmetry [2]. Patients with PCD cannot clear mucosal buildup and are classically prone to developing recurrent upper and lower respiratory tract infections, chronic rhinosinusitis, progressive bronchiectasis, and atelectasis [3].

Genetic mutations in more than 50 genes can lead to a spectrum of phenotypic expression in PCD [4]. Furthermore, mutations in genes encoding for the radial spoke head (RSPH) proteins are known to be involved in ciliary dysfunction and PCD pathogenesis [5]. The radial spoke head protein 4 homolog A (*RSPH4A)* gene has been described as having an important role in the assembly of radial spokes [6]. This article summarizes the current knowledge about the *RSPH4A* gene in PCD. We cover everything from genetic mutations and cilia dysfunction to animal model organism orthologs and human clinical phenotypes.

## 2. The *RSPH4A* Gene and Protein in Ciliary Dysfunction

**Figure 1:**
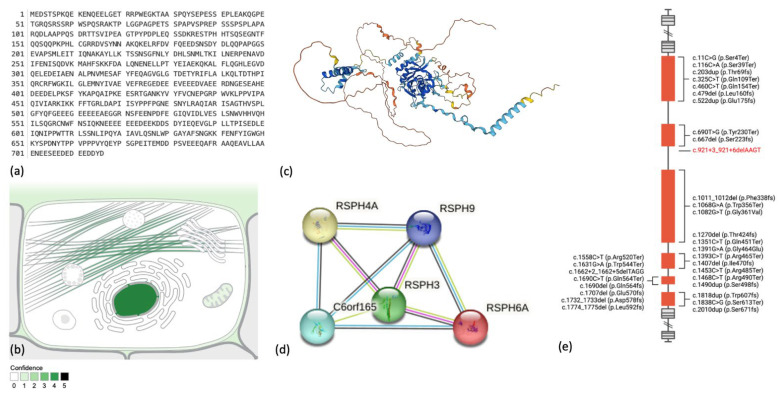
The *Homo sapiens* (human) radial spoke head protein 4 homolog A (RSPH4A) protein (Q5TD94). (**a**) Complete amino acid sequence. (**b**) Subcellular localization from the COMPARTMENTS database. (**c**) Three-dimensional AlphaFold structure prediction. The per-residue confidence score is represented in colors (**d**) The protein-to-protein association network from the STRING database. Lines colors represent known and predicted interaction between proteins (**e**) *RSPH4A* gene exon structure and disease-causing variants; red font identify the Puerto Rican founder mutation *RSPH4A* (c.921+3_921+6delAAGT (intronic)). Figure 1e was created with BioRender.com (accessed on 24 November 2022).

The *RSPH4A* gene is located on chromosome 6q22.1 (chr6:116,616,479-116,632,985 (GRCh38/hg38)). There are 16,507 nucleotides in the *RSPH4A* gene. The gene’s transcription and translation process results in a protein with a molecular mass of 80,733 Daltons and 716 amino acids [7]. The nucleus and cytoskeleton are the most common subcellular locations for the RSPH4A protein [8], but its presence at the mitochondrion, plasma membrane, and extracellular matrix have been observed. Protein-to-protein interactions exist between RSPH4A and RSPH9, RSPH3, and PRSPH6A, and C6orf16 (Figure 1) [9]. Several disease-causing pathogenic variants have been described in humans with a clinical phenotype of PCD [10]. Figure 1e depicts these pathogenic variants in the *RSPH4A* gene associated with PCD.

Motile cilia are structured in a (9 + 2) microtubule doublet pattern consisting of nine peripheral outer doublets connected to a central complex or in a (9 + 0) arrangement with the absence of the central complex [11]. The (9 + 2) structural arrangement of motile cilia is usually found in the upper and lower respiratory tracts, spermatozoa, and lining of the female reproductive tract [12]. A (9 + 0) structural arrangement in motile cilia, also known as nodal cilia, is essential in determining body left-to-right organ asymmetry during embryological development [13]. Ciliary ultrastructure is directly affected by *RSPH4A* mutations due to the crucial role of RSPH proteins in ciliary stability and the interconnections between peripheral outer doublets and the central complex [14]. Radial spoke proteins interact with dynein arms, and the central complex governs important aspects of the ciliary movement [15,16]. Alterations in ciliary ultrastructure and cilia motility are important contributors to impaired ciliary function and the progression of clinical phenotypes affecting pulmonary function and fertility [17,18]. The wide array of genetic and protein alterations contributing to the changes in the ciliary ultrastructure is an intriguing and challenging spectrum of genotype-phenotype manifestations in humans [19].

## 3. Homologs for the *RSPH4A* Gene—Animal Models

**Figure 2:**
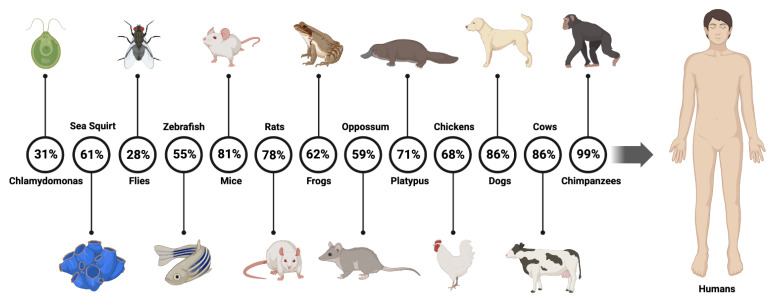
Animal model and orthologs similarity for the human *RSPH4A* gene. Percentage of the *RSPH4A* human gene sequence matching the sequence of the orthologs. Created with BioRender.com. Data obtained from GeneCards—the human gene database [7].

The *RSPH4A* gene is conserved across animal species and animal model findings [20]. Throughout evolution, a few RSPH protein-coding genes have persisted across many species [21]. The literature has shown that the *RSPH4A* gene is conserved in *Chlamydomonas* spp., sea squirt, flies, zebrafish, mice, rats, frogs, opossums, platypus, chickens, dogs, cows, and chimpanzees [5], for a total of 200 species with a human *RSPH4A* ortholog [7]. Figure 2 summarizes the similarities between different organisms and humans.

The protein composition of Chlamydomonas radial spokes was determined, as were the positions of the various subunits within this structure [22]. Twelve of the 23 radial spoke proteins found in Chlamydomonas appear to have human orthologs, and mutations in these genes are likely to result in the malfunctioning of motile cilia [20]. It has been studied that both *RSPH9* and *RSPH4A* encode radial spoke head proteins based on homology with proteins with known activities in the biflagellate alga *Chlamydomonas reinhardtii* and other ciliates. The RSPH9 protein from the biflagellate alga *Chlamydomonas reinhardtii* is proven to be 28% identical to the human *RSP9* protein [23]. The RSP4 protein from *Chlamydomonas* spp. was 31% similar to the RSPH4A protein in humans [5]. The central complex is lost in human motile cilia with *RSPH4A* truncation mutations, whereas the central complex is preserved but shifted in *Chlamydomonas* spp. pf17 with an *RSP9* truncation mutation [24]. A mutation in RSP4 results in flagella immobility [14]. Due to the structural differences of the radial spoke, there are limitations to the use of *Chlamydomonas* spp. as a model for PCD in humans.

A murine model was used to investigate the role of the RSPH4A protein and how mutations in the *RSPH4A* gene affect the cilia assembly [25]. Although the phenotypes differ, it has been established that the RSPH4A protein regulates the motion pattern of mouse motile cilia. The RSPH4A building block is shared by all three triplet spoke heads: RS1, RS2, and RS3. In contrast to humans, where RS3 is preserved, all three types of spoke heads are absent in *RSPH4A* knockout mice, indicating that *RSPH4A* is essential for assembling the triplet spoke heads. The lack of all triplet spoke heads in *RSPH4A* knockout mice may explain the more severe structural abnormalities of the axoneme of the respiratory cilia than in *RSPH4A* patients. The finding that only 50% of respiratory cilia in human *RSPH4A* patients exhibit normal axonemal structure, compared to 80% in *RSPH1* patients, suggests that the *RSPH4A* mutation causes a more severe phenotype than the *RSPH1* mutation [6]. Wang et al. investigate how cilia abnormalities in animal models with a human ortholog may be a potential cause of infertility in people with *RSPH4A* gene pathogenic variants. Wang et al. looked at infertility caused by *RSPH4A* gene variants in three Chinese families and showed that female patients with *RSPH4A* pathogenic variants may have infertility due to an abnormal pattern of oviduct cilia beating [17]. They had previously observed that the oviduct cilia in *RSPH4A* mutant mice displayed two abnormal motion patterns: anti-clockwise rotation and small amplitude beating [6].

## 4. *RSPH4A* Gene—Clinical Phenotype in Humans

### 4.1. Pulmonary Disease in Humans with the RSPH4A Mutation

**Figure 3:**
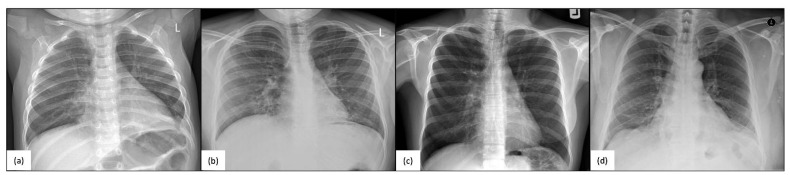
Chest X-rays (CXR) of homozygous patients with PCD for the *RSPH4A* (c.921+3_921+6delAAGT (intronic)) founder mutation. (**a**) A one-year-old male showing air trapping, (**b**) a 13-year-old female with perihilar infiltrates, (**c**) a 38-year-old female with bibasilar atelectasis, and (**d**) a 51-year-old male with worsening bibasilar atelectasis and fibrotic tissue. No laterality defects are evident. Panel organized using BioRender.com (accessed on 24 November 2022).

**Figure 4:**
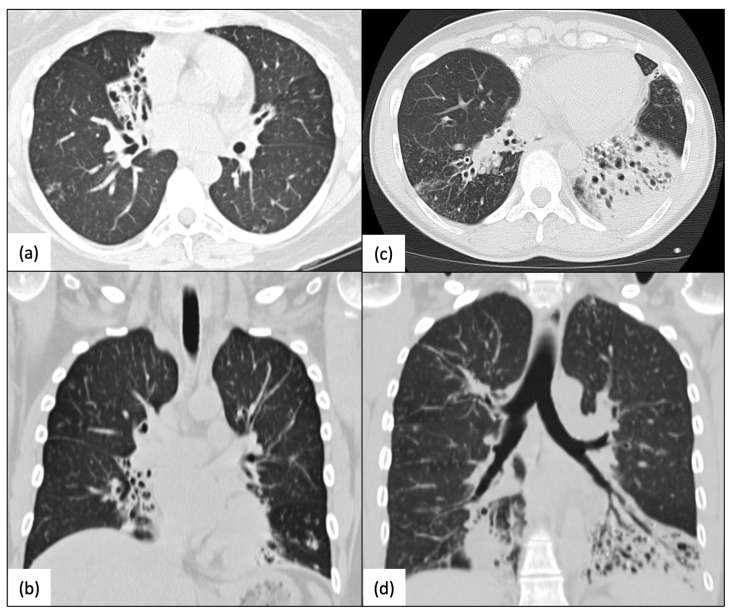
High-resolution CT scans (HRCT) of the chest. Homozygous patients with PCD for the *RSPH4A* (c.921+3_921+6delAAGT (intronic)) founder mutation. (**a**,**b**) A 40-year-old female with mild-to-moderate RML and LLL varicose bronchiectasis with a bilateral and peripheral tree-in-bud pattern, and (**c**,**d**) a 59-year-old female with increased bronchovascular markings, severe bibasilar bronchiectasis, mucus plugs, and atelectasis, more prominent at LLL. Panel organized using BioRender.com (accessed on 24 November 2022).

Pathogenic variants in *RSPH4A* cause changes in ciliary ultrastructure, primarily disrupting the ciliary beating pattern and resulting in a lack of adequate mucociliary clearance, which is a significant contributor to the development of a clinical PCD phenotype [17]. Patients with confirmed *RSPH4A* genetic variants have a year-round wet cough, chronic nasal congestion, neonatal respiratory distress, bronchiectasis, chronic secretory otitis media, and hearing loss [26,27]. Chest X-rays of *RSPH4A* PCD patients, both pediatric and adult, show atelectasis and perihilar infiltrates (Figure 3). Cylindrical varicose bilateral bronchiectasis, bibasilar parenchymal scarring tissue, centrilobular nodules, and tree-in-bud opacities are common characteristics found on a high-resolution CT (HRCT) scan of the chest in individuals with *RSPH4A* PCD, as depicted in Figure 4.

### 4.2. Pulmonary Function in PCD with RSPH4A Pathogenic Variants

**Figure 5:**
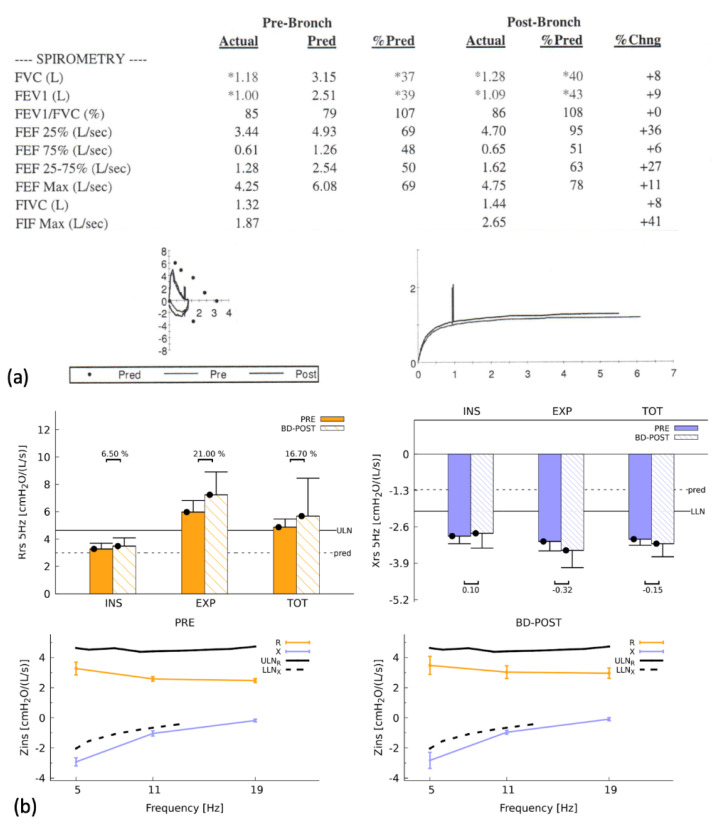
Spirometry and forced oscillation technique (FOT) of a homozygous PCD patient with the *RSPH4A* (c.921+3_921+6delAAGT (intronic)) founder mutation. (**a**) Airflow limitation with a restrictive pattern is observed in basic spirometry. The flow-volume and time-volume loops are abnormal, with no significant improvement after the bronchodilator. (*) shows values below the lower limit of normal. (**b**) FOT revealed the presence of respiratory impairment without significant reversibility. Respiratory impedance (Zins) was consistent with severe obstructive disease. Panel organized using BioRender.com (accessed on 24 November 2022).

The sino-pulmonary pathophysiology is markedly similar to that of other PCD-causing variants. Mucus is not effectively removed from the respiratory airways in individuals with PCD due to a lack of appropriate ciliary function [28], increasing the risk of recurrent pulmonary infections [29] and eventually leading to chronic lung disease and alterations in pulmonary function [30]. Halbeisen et al. demonstrated that PCD patients had lower mean Forced Expiratory Volume in One Second (FEV1) and Forced Vital Capacity (FVC) measurements [31]. A more detailed longitudinal observational study found that FEV1 reduction was associated with ciliary ultrastructural abnormalities and genotypes in PCD patients [32]. A study also found that the patient’s ciliary ultrastructure deficiencies and genotype have an impact on lung function progression [33]. PCD patients exhibit impaired lung function compared to reference values, and lung function indices can be linked to genotypes and ciliary ultrastructure anomalies. Figure 5 depicts pulmonary function as well as the results of the forced oscillation technique in an *RSPH4A* PCD patient.

The *RSPH4A* genotype exhibits a wide range of microtubular disorganization patterns and central complex abnormalities, which influence the clinical presentations. Such ciliary ultrastructure defects affect the pulmonary function profile in *RSPH4A* patients differently than other PCD genotypic variations. Complete pulmonary function in *RSPH4A* patients is typically characterized by a severe obstructive pattern manifested by a large decrease in FEV1 and significant air trapping as indicated by elevated residual volume (RV) and specific airway resistance parameters (sRaw) [27]. Concomitant comorbidities like bronchial asthma may influence these findings.

### 4.3. Absence of Laterality Defects

A key distinguishing observation is that patients with *RSPH4A* pathogenic variants show no laterality defects [17,26,27]. This observation is supported by the fact that throughout embryonic development, nodal cilia are primarily responsible for determining the body’s left-to-right asymmetry [13]. Because nodal cilia lack a central apparatus and have a [9 + 0] microtubule configuration, *RSPH4A* pathogenic variants affect the radial spoke proteins and have no effect on the left-to-right axis. The nodal cilia in *RSPH4A* PCD patients operate normally throughout embryonic development.

## 5. Diagnostic Findings for *RSPH4A* PCD Patients

### 5.1. Transmission Electron Microscopy (TEM)

**Figure 6:**
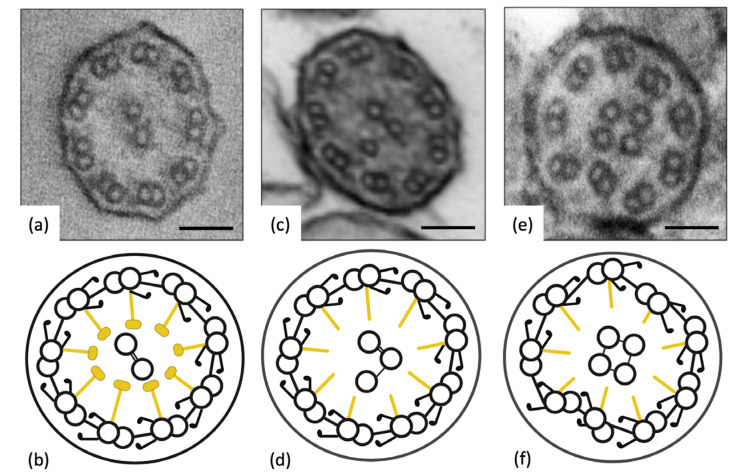
Transmission Electron Microscopy (TEM). Ciliary biopsies of patients with the *RSPH4A* (c.921+3_921+6del (intronic)) founder mutation. (**a**,**b**) Normal, healthy subject. (**c**,**d**) Abnormal central complex microtubule configuration, (9 + 3). (**e**,**f**) Abnormal peripheral and central microtubule organization and configuration, (9 + 4). Scale bar for (**a**,**c**,**e**): 50 nm. Illustrations (**b**,**d**,**f**) were created with BioRender.com (accessed on 24 November 2022).

TEM analysis of ciliary ultrastructure in *RSPH4A* gene variants reveals abnormal findings in approximately 50% of the cilia’s ultrastructure (Figure 6). The primary ciliary defects are the absence of the central complex and changes in microtubule organization, as per international consensus guidelines [34].

Some studies have reported a transposition defect in which TEM imaging of cilia cross-sections revealed an absent central pair (9 + 0). Others display an (8 + 1) arrangement in 10% of the cases, arguing for a peripheral microtubule doublet being transposed to the central complex [35,36]. A TEM examination of 11 *RSPH4A* cilia biopsies from Puerto Rican individuals confirmed by genetic testing revealed abnormal findings [27]. These findings were linked to defects in the central apparatus and abnormal microtubule conformation, loss of central and outer doublets, a solitary central microtubule, and an increase in the number of central singlets, triplets, and quadruplets [37].

The limitations of TEM in diagnosing *RSPH4A* include the misinterpretation of secondary ultrastructural defects in cilia caused by acute or chronic respiratory infections, inflammatory respiratory diseases such as viral infections, environmental factors, demographics such as age, or sample handling [34,38]. Misinterpreting ciliary defects is avoided by collecting samples while the subject is healthy or by using cell cultures to eliminate the possibility of secondary defects in *RSPH4A* cilia analysis [39]. Individual processing techniques and familiarity with the local appearance of the cilia are critical in understanding the cilia ultrastructure of *RSPH4A* and other PCD genes. Differences in equipment, availability and local procedures or resources make it difficult to standardize sample processing methodology; thus, a consensus methodology is recommended [40]. Given the particularities of the *RSPH4A* TEM defect, it is necessary to evaluate more than one sample or cell culture and incorporate additional tests in the diagnosis of the ciliary ultrastructure, such as high-speed video microscopy analysis (HSVA), nNO measurements, immunofluorescence, and genotyping, for an accurate PCD diagnosis. Nevertheless, TEM is a helpful ancillary diagnostic test and can be used to visually characterize PCD-causing variants.

### 5.2. Nasal Nitric Oxide (nNO)

**Figure 7:**
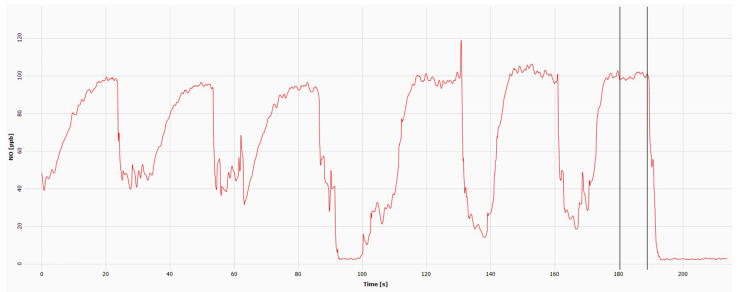
Chemiluminescence detection of nasal nitric oxide (nNO) in a PCD patient homozygous for the *RSPH4A* (c.921+3_921+6delAAGT (intronic)) founder mutation. Time-lapse from 0 to 90 s: left nostril; 90–200 s: right nostril. The average nNO using the exhalation against resistance technique was 34 nL/min (cutoff < 77 mL/min). ppb = parts per billion. The mean concentration of the right and left nares together times the flow sampling rate (L/min) equals the final nNO value (nL/min).

Nitric oxide (NO) is a signaling molecule synthesized in the respiratory epithelium in response to inflammation or infections [41]. Studies on PCD patients have shown that nNO levels are significantly lower than those of healthy control subjects [42]. Testing nNO levels is safe, noninvasive, feasible, and accurate in diagnosing PCD [43]. However, nNO testing in PCD is expensive and requires specialized equipment, trained personnel, and standardized operating protocols to be successfully implemented in fully accredited PCD centers. Previous studies have shown that individuals with *RSPH4A* have low nNO levels, less than 77 nL/min [26,44]. Daniels ML et al. reported an average of 10–42.7 nL/min. Similarly, De Jesús-Rojas et al. found that biallelic homozygous patients with the *RSPH4A* (c.921+3_921+6delAAGT (intronic)) founder mutation had median nNO levels of 19.9 nL/min by chemiluminescence technology [44]. Some reports have found *RSPH4A* PCD patients with values above 77 nL/min [45]. Figure 7 presents an example of an nNO measurement using the EcoPhysics (CLD 88sp Chemiluminescence Nitric Oxide Analyzer, Dürnten, Switzerland). In cooperative patients with clinical suspicion over five years, the nNO diagnostic tool is sensitive and specific for PCD. Analytical studies have confirmed that nNO is as accurate as TEM or genetic testing in patients with a high clinical suspicion of PCD who have had cystic fibrosis ruled out by a sweat test [42].

### 5.3. High-Speed Video Microscopy Analysis (HSVA)

HSVA is a technique that uses light microscopy to observe respiratory cilia ex vivo and a high-speed video camera to record them [46]. Ciliary beat frequency (CBF), ciliary beat pattern (CBP), and effective mucociliary clearance are all measured in recorded videos [47]. As a diagnostic tool, HSVA allows the ciliary function to be tested on the same day [48]. Given the time and resources required for HSVA, it is the only diagnostic tool to visualize and analyze living respiratory cells with beating cilia in vitro with quick and cost-effective results from experts on the technique [49]. In contrast, TEM and genetic analysis can provide results over weeks or months. When TEM is not feasible due to the absence of morphological changes, HSVA provides an additional tool for PCD diagnosis. This technique is more commonly used in PCD centers in Europe and Australia, and some centers in North America are beginning to implement it [50]. Previous studies on HSVA have demonstrated high sensitivity and specificity as a PCD diagnostic test, making it a reliable test while confirmatory studies are conducted [48]. Previous HSVA studies on *RSPH4A* individuals revealed a slightly slower CBF (4.7 ± 1.2 Hz) in random regions [26]. On samples evaluated from the lateral perspective, there was a decreased range of motion and a lack of coordinated movement with surrounding cilia. The *RSPH4A* cilia showed “rotational” movement, like other *RSPH* genes reported in PCD [6,26]. Further research and characterization of the HSVA of individuals with *RSPH4A* variants are required.

### 5.4. Immunofluorescence in RSPH4A

Immunofluorescence is highly specific and increases the speed and accessibility of PCD testing. It is not recommended as a stand-alone test for diagnosing PCD due to its low sensitivity [51]. The main advantages of this test are its simplicity, widespread availability, and low cost. Immunofluorescence analysis reduces the need for repeated patient biopsies, improving turnaround time while maintaining diagnostic accuracy [51]. Using specific and commercially available antibodies with fluorescent tags, the immunofluorescence technique allows for the indirect imaging of cilia-targeted proteins using fluorescent or confocal microscopy to confirm protein absence due to PCD genetic mutations. The co-localization of ciliary proteins can be determined by using different double-labeling tags. Many antibodies for proteins involved in PCD have been commercialized and validated by experts, including RSPH4A [52]. The RSPH4A protein is the core protein of the radial spoke head in ciliary axonemes and may be detectable using immunofluorescence [53]. A significant limitation of this technique is that antibodies are exclusively used in specific proteins of interest, leaving defects in unrelated proteins unrecognized [51]. Because most genetic variants affecting the motile cilia’s radial spoke, such as *RSPH4A*, are loss-of-function mutations, the immunofluorescence technique can be used as a complementary ancillary tool to understand disease-causing genes and their relationship to ciliary structure [53]. Because diagnostic tools for the radial spoke structure, such as TEM and HSVA, can produce normal results, immunofluorescence is a valuable diagnostic tool that increases specificity and, thus, is a valuable resource to have in challenging cases of *RSPH4A* diagnosis. The addition of immunofluorescence analysis into the diagnostic algorithm for *RSPH4A* is recommended due to its enhanced diagnostic accuracy and reduction of further biopsies in patients with clinical suspicion of *RSPH4A* pathogenic variants in PCD [54].

## 6. Ancestry of *RSPH4A* Founder Puerto Rican Mutation

The average Puerto Rican genetic composition includes 64% European, 21% African ancestral, and 15% Native American Taino descent [55]. Because of Puerto Rico’s prominent Taino ancestry, the *RSPH4A* (c.921+3_921+6delAAGT (intronic)) founder mutation was considered to be endemic to the island. An original hypothesis attributed the origin of the founder mutation to Taino ancestry due to the presumed prevalence of recessive conditions inherited through mitochondrial genes as well as the geographical isolation of the favored island [26]. Nevertheless, a median-joining haplotype network built from the genome sequences of 104 Puerto Rican subjects in the 1000 Genomes Project discovered two subjects with the *RSPH4A* (c.921+3_921+6delAAGT (intronic)) founder mutation. Local ancestry analysis determined both chromosomal segments of the *RSPH4A* gene to be of European origin [56]. These results suggest that the *RSPH4A* (c.921+3_921+6delAAGT) splice site mutation may have been carried to Puerto Rico by Europeans shortly after their arrival, resulting in an increased frequency due to genetic drift. Additional studies are needed to determine the ancestry of other *RSPH4A* pathogenic variants in specific population groups.

## 7. The *RSPH4A* Gene in the Past 5 Years

Since the *RSPH4A* gene was sequenced fifteen years ago, our understanding of the role of *RSPH4A* pathogenic variants in PCD has enhanced our medical knowledge about this rare disease. More than 20 peer-reviewed articles on the *RSPH4A* gene have been published in the last five years. In 2018, Han et al. described the role of the radial spoke protein as essential in ciliary motility during early zebrafish embryonic development [57]. Takeuchi et al. described a Japanese family with a novel *RSPH4A* pathogenic variant as part of 46 patients with suspected PCD in a prospective study [58]. In 2019, Emiralioğlu et al. demonstrated the genotype-phenotype relationship of five non-consanguineous Turkish patients with *RSPH4A* pathogenic variants and considerable lung disease heterogeneity [59]. Yoke et al. published one of the most significant studies in 2020, revealing the function of the radial spoke domains RS1, RS2, and RS3 in mice motile cilia as an explanation for the severe PCD phenotype in humans [6]. Zhao et al. published a follow-up article on the human *RSPH4A* gene in 2021, supporting previous findings by validating the *RSPH4A* structure in a three-dimensional model using cryo-electron tomography [14]. In 2021, our group published eight papers, including the confirmation of an *RSPH4A* (c.921+3_921+6delAAGT (intronic)) founder mutation in Puerto Rico and their ancestral heritage [27,56]. The same year, Bian et al. presented an intriguing case report describing a patient with concurrent neurofibromatosis and PCD caused by a homozygous *RSPH4A* pathogenic mutation (c.667delA, p.S223Afs*15) [60]. In 2022, the discovery of novel pathogenic variants (c.2T>C, p.(Met1Thr); c.1774 1775del, p.(Leu592Aspfs*5)) associated with PCD-related infertility in Chinese families was linked to asthenoteratozoospermia [17]. More recently, our group published a detailed description of the *RSPH4A* pathogenic variant dispersed geographically in Puerto Rico and nNO levels in a similar cohort of patients with *RSPH4A*-related PCD [44,61]. Even though knowledge of the *RSPH4A* gene is strongly associated with PCD, further research is needed to understand the long-term clinical implications of pathogenic variants in *RSPH4A* on the human spectrum of PCD.

## 8. Conclusions

The genetic profile of the patient influences the phenotypic variability of PCD patients. The gene and protein synthesis produce a structure that plays an important role in the normal physiology of the cilia. The *RSPH4A* gene is conserved throughout evolution from unicellular to multicellular organisms. Pathogenic variants in the *RSPH4A* gene generate a multisystemic disease with multiple comorbidities resulting from chronic oto-sino-pulmonary infections. The lack of laterality defects makes diagnosing *RSPH4A* PCD patients challenging. Following clinical suspicion, ancillary tests such as TEM, nNO level measurement, HSVA, and immunofluorescence guide the clinician to a confirmed PCD diagnosis. Because no single test is considered the gold standard for PCD, research into new screening tools to identify patients at birth is required. The implementation of a PCD newborn screening test in countries with an increased prevalence of a specific PCD mutation may be a possibility in the near future.

## Data Availability

Not applicable.
